# Supplementary material for: HNRNPH1-mediated splicing events regulate EIF4G1 transcript variant composition and the organization of the AURKA 5’UTR
Source: bioRxiv. 2025 Jul 29:2025.07.28.667222. Preprint. [Version 1] doi: 10.1101/2025.07.28.667222 (PMC12324280; doi:10.1101/2025.07.28.667222)
Supplement: Supplement 1 [file media-1.pdf]

SUPPLEMENTARY MATERIAL

HNRNPH1-mediated splicing events regulate *EIF4G1* transcript variant composition and the organization of the *AURKA* 5′UTR

Tayvia Brownmiller[1], Soumya Sundara Rajan[1], Tamara Jones[1], Patricio Pichling[1], Vernon J. Ebegboni[1], Ashish Lal[4], Ioannis Grammatikakis[4], Erica C. Pehrsson[2,3], Natasha J. Caplen[1]*

Supplementary Table S1. Reagents and Resources

Supplementary Table S2. rMATS analysis, the prioritization of genes for further analysis, TCF3 LSVs and differential transcript expression (EB-seq) (Excel file)

Supplementary Table S3. Summary of MYCN and MYC peaks (Excel file)

Supplementary Table S4. Gene-specific outputs of long read RNA sequencing (PacBio, Iso-seq), detected LSVs, and differential transcript expression (EB-seq) (Excel file)

Supplementary Figure S1. HNRNPH1's transcriptome-wide regulation of alternative splicing

Supplementary Figure S2: HNRNPH1's regulation of a *TCF3* mutually exclusive splicing event

Supplementary Figure S3: The differential expression of *TCF3* transcript variants in neuroblastoma

Supplementary Figure S4: HNRNPH1 regulates the inclusion of *NUMB* exon 12 and usage of the *EIF4G1* exon 4-5 junction

Supplementary Figure S5: HNRNPH1-regulated splicing events affecting *NUMB* and *EIF4G1* transcripts

Supplementary Figure S6: HNRNPH1's regulation of an *AURKA* 5′UTR skipped exon (SE) event and *AURKA* RNA stability

Supplementary Table S1

Cell lines	Source	STR date
HEK-293T	ATCC, Manassas, VA	December 2023
HT-1080	ATCC, Manassas, VA	July 2023
SK-N-FI	Genetics Branch, CCR NCI	November 2024
IMR-32	Genetics Branch, CCR NCI	November 2024

Primers	Source	Sequence	Comment
HNRNPH1 forward	Invitrogen (Thermo Fisher Scientific), Waltham, MA	5′-TACACATGCGGGGATTACCTT-3	Neckles *et al* (2019) (1)
HNRNPH1 reverse	Invitrogen (Thermo Fisher Scientific)	5′-CTTCACCAGTTACTCTGCCATC-3'	
NACA forward	Invitrogen (Thermo Fisher Scientific)	5′-ACAAGAGCCCTGCTTCAGAT-3′	
NACA reverse	Invitrogen (Thermo Fisher Scientific)	5′-GCTGCTAGTTGTGCTTGCTG-3′	
RPL27 forward	Invitrogen (Thermo Fisher Scientific)	5'-GCAAGAAGAAGATCGCCAAG-3'	
RPL27 reverse	Invitrogen (Thermo Fisher Scientific)	5'-GACGACAGTTTTGTCCAAGG-3'	
TCF3-exon 17 forward	Invitrogen (Thermo Fisher Scientific)	5'-AGGAGAAGGAGGACGAGGAG-3'	Yamazaki *et al* (2018) (2)
TCF3-exon 19 reverse	Invitrogen (Thermo Fisher Scientific)	5'-GGAGCTGAAAGCACCATCTG-3'	
EIF4G1-exon 1 forward	Invitrogen (Thermo Fisher Scientific)	5'-CGCGGACTGAAGGAGACTGA-3'	
EIF4G1-exon 5 reverse	Invitrogen (Thermo Fisher Scientific)	5'-AGCAGGGTAGACATGGGCAG-3'	Gonatopoulos-Pournatzis *et al* (2020) (3)
EIF4G1-exon 5 reverse	Invitrogen (Thermo Fisher Scientific)	5'-AGCAGGGTAGACATGGGCAG-3'	
AURKA-exon I/III forward	Invitrogen (Thermo Fisher Scientific)	5'-TCCTTGGGTCGCAGGCT-3'	Also used for *AURKA* minigene
AURKA-exon I/II (~100nt) forward	Invitrogen (Thermo Fisher Scientific)	5'-TCCTTGGGTCGCAGGCT-3'	
AURKA-exon I/II (>100nt) forward	Invitrogen (Thermo Fisher Scientific)	5'-CAGCTAGAGGGTCTCACTCC-3'	
AURKA transcript variant reverse	Invitrogen (Thermo Fisher Scientific)	5'-GCCTTAACAGGTCCTGAAATGC-3'	
18s forward	Invitrogen (Thermo Fisher Scientific)	5'-GTAACCCGTTGAACCCCATT-3'	
18s reverse	Invitrogen (Thermo Fisher Scientific)	5'-GGACTTAATCAACGCAAGCT-3'	
RTRHC Reverse (cDNA synthesis)	Invitrogen (Thermo Fisher Scientific)	5'-GAGCTTTGCAGCAACAGTAAC-3'	Singh and Cooper (2006) (4)
AURKA-exon Ia forward	Invitrogen (Thermo Fisher Scientific)	5'-TTGGGTCGCAGGCATCATG-3'	
AURKA-exon Ia-II forward	Invitrogen (Thermo Fisher Scientific)	same as *AURKA*-exon I/II (~100nt)	
AURKA-exon Ia-III forward	Invitrogen (Thermo Fisher Scientific)	same as *AURKA*-exon I/III	
AURKA-exon Ib forward	Invitrogen (Thermo Fisher Scientific)	5'-ACGGGCATCATGGACCGAT-3'	
AURKA-exon Ib-II forward	Invitrogen (Thermo Fisher Scientific)	5'-GACGGGGTCTCACTCCATT-3'	

AURKA-exon Ib-III forward	Invitrogen (Thermo Fisher Scientific)	5'-GACGGGCTGGAGTGCAAT-3'	
AURKA-exon Ic forward	Invitrogen (Thermo Fisher Scientific)	5'-CAGCTAGAGGCATCATGGACC-3'	
AURKA-exon Ic-II forward	Invitrogen (Thermo Fisher Scientific)	same as AURKA-exon I/II (>100nt)	
AURKA-exon Ic-III forward	Invitrogen (Thermo Fisher Scientific)	5'-CTAGAGGCTGGAGTGCAATGG-3'	
RTRHC Reverse (minigene)	Invitrogen (Thermo Fisher Scientific)	5'-GGAATATGCACCACTTGGAACA-3'	
AURKA (qPCR) forward	Invitrogen (Thermo Fisher Scientific)	5'-GGAATATGCACCACTTGGAACA-3'	CDS-exon 5
AURKA (qPCR) reverse	Invitrogen (Thermo Fisher Scientific)	5'-TAAGACAGGGCATTTGCCAAT-3'	CDS-exon 6
GAPDH forward	Invitrogen (Thermo Fisher Scientific)	5'-TGCACCACCAACTGCTTAGC-3'	Actinomycin-D qPCR
GAPDH reverse	Invitrogen (Thermo Fisher Scientific)	5'-GGCATGGACTGTGGTCATGAG-3'	

**PCR/qPCR Reagents**	**Company**	**Catalog #**
5X iScript RT Supermix (cDNA synthesis)	Bio-Rad Laboratories, Hercules, CA	1708841
SuperScript II Reverse Transcriptase (cDNA synthesis)	Invitrogen (Thermo Fisher Scientific)	18064-014
Q5 Hot Start High-Fidelity 2X Master mix	New England BioLabs, Ipswich, MA	M0494S
Phusion High-Fidelity PCR Master mix with GC Buffer	New England BioLabs	M0532S
PowerUp SYBR Green Master Mix	Applied Biosystems (Thermo Fisher Scientific)	A25742
TrackIt 100bp DNA ladder	Invitrogen (Thermo Fisher Scientific)	10488058
6% TBE Gel	Invitrogen (Thermo Fisher Scientific)	EC6265BOX
Novex Hi-Density TBE Sample Buffer (5X)	Invitrogen (Thermo Fisher Scientific)	LC6678
100bp DNA Ladder	Invitrogen (Thermo Fisher Scientific)	15628019
SYBR Safe DNA Gel Stain	Invitrogen (Thermo Fisher Scientific)	S33102

**Reporter plasmids and DNA cassettes**	**Source**	**Catalog number or sequence**
pRHCglo	Addgene, Watertown, MA	80169; Singh and Cooper (2006)
AURKA-5'UTR DNA cassette	Custom synthesis Genewiz, South Painfield, NJ<br><br>Restriction enzymes: **SalI (gtcgac)** and **SpeI (actagt)**	**gtcgac**ggttcttagggagcaagtgcgcctgcgcgcggtgtgcgcccttaaacgcgactcaaggcgt<br>cgggtttgttgtcaaccaatcacaaggcagcctcgctcgagcgcaggccaatcggctttctagctagag<br>ggtttaactcctatttaaaaagaagaacctttgaattctaacggctgagctcttggaagacttgggtcctt<br>gggtcgcaggtgggagccgacgggtgggtagaccgtgggggatatctcagtggcggacgaggac<br>ggcggggacaaggggcggctggtcggagtggcggagcgtcaagtcccctgtcggttcctccgtccc<br>tgagtgtccttggcgctgccttgtgcccgcccagcgcctttgcatccgctcctgggcaccgaggcgccc<br>tgtaggatactgcttgttacttattacagctagaggtacaaggggtttgttgagtggtgttgacatgcgc<br>gggaggggtgggtgggcttcagattggattttgtcctccgagatcacctgggtaagaaaagcacaga<br>aggtctctgctgtgttggagttcacgttctagtgggtggggagacagacggtaagcgtacggagaac<br>ttgcagctggggtgggtgttacagaggaaaagcaggagtgcggtttaacgggggccgctttagata<br>gaatagcctaagaaggcccttgtcctggctggatgagtgggtgaattgatgaatgagaacctccttgc<br>agaggccttcccggtccgtacttctcagaccgtagaccttggagcagttgtaccgggataacttgcagt<br>gcttgttaaacatatagctccaccgagagcccggtaaatcagaatctctgaggggtaggacccaggtg<br>tctagttattaacaaactctccagatgattcttaggtttcattgagaactagggatgcagaccggtgcata<br>caaatcgtctggggacgttaaaatgcagaatctgtttttttagacctggggttaggcctcagagtctccat<br>ttctaagaaccatccggctgaggtagacgtagccactgtccttaactctcgtaatgtctcttcctcttccgt<br>aaccttccttgtcccttgaattaaacgtttttcagcaacctactcagttcgtccttcccttcatctctgcagaca

		tgcacaggtctgagggaggaaggaataaaccgtataaacctcctgcgctattagcctaacagcttttct<br>attcaaaatagtaggacttctggtttgaactgaatggatcctgtgaaagtcatctggttcttcaatctggtt<br>gcagattagagtcacctgaagggcagtctccttggcgttgtctccagaattctggattagaatcttattcc<br>attctgcttgttattcaatttccctagaaagaaaggtagaataaattggagcaaatgcctgtagcttctgtc<br>agaagaatgttgaataaatgttgttaggcctatgtgatctcattagactgctacttagaattgtaaggga<br>agtaaagcattagagcatgtgtgaaattaaatatttgattaacacaagtgtgcatttccttgttgctgtttat<br>caacttttacttacccactgtttttttataagggctgcagcctgtagtctgggcctggcttcatcatggaatt<br>atttgcttaattgtaaaatggtaatcttaattttttttttttttgagacagggtctcactccattgcccaggccaga<br>gtgcggggatatttgataagaaacttcagtgaaggccgggcgcggtggctcatgcccgtaatcccag<br>cattttcggaggccgaggtgggtggatcacctgaggtcgggagttccagaccagccctaccaacata<br>gagaaaccctgtctctactaaaaatacaaaatcagctgggcgtggtggtgcatgtctgtaatcccagct<br>actctggaggctgaggcaggagaattgcttgaacccaggaggtggaggttgcgggtgagccgaga<br>ttgcgccattgcactccagcctgggcaacaagagcaaactctatctcaaaaaaaaaaaaaaagaaacct<br>cagtgaaatgaaagactgaactacacacctgggggaaaagcgtaataaagggagcaagaagtgca<br>aatgcttatggcaggggacttatttgcagaaagaaagccttgtgtggctggtacacggttgacaagaa<br>gaggagatgaagtcagagaggtagttagggtccatgttctgtgagacttcaataggcctgtctatgca<br>ggttaggagtttgcattgttttgattgagttgggagccatggggggtcttctgaactgaggatcaacacat<br>ctgatttgttaatgagattgccggcctcagtgttaagaatggaccatgacagggtaagggtggaagtg<br>gggaggccattgggaggctgttggaaaaaactcaagggagagagaataatggtttgaccaggtggt<br>agtagtagaggtggtgagcagtgatcttgaaggcagagcctacagaatatgctggtggattaaatga<br>agggtttgcgagagagaacctcgtaaagggttattatgccaaggcttttggcctgagcaactgacagg<br>atgggtttgccatgcactgagatgaggatgtctagagactagatttggggaggtgagagtggggag<br>agattaggacttctgtttgggacatgttagaatggagatgccgctcagataatcaggtgcagatgttgc<br>atagaaagtagaaaatgtagatcaagagttcaggggagaggtctgggctgaagaatgaatatttggg<br>agtcctcagcctgtagatggtactgaaagcaaagagactggatgtactcggctagggagtaattgga<br>gatggagaagtcattctaggcctgagctcagggcacacccatgaacagggaggtgagaagtggag<br>aagggaagaggttcagttttggatatgttaggattgaggtgcctggcagacatttttgggagatcctgg<br>gcaaatccttggatatccaagtctgacagtccaaggagagactagggcagaagacgcactttgagtg<br>gtcctcatgaaggcatttagagctgaggcactgggtgatatggatccctggtgaaggtgtagatgga<br>gaagtggagggtccagggactcgttcttgagtcaccccagacccttgatctggcaaaaatgagactga<br>cccagcaaggagggaaagacaggacgactaggtggtagataaacctggaagtgaagtgaagaaa<br>aaagtgtaagatgtgatcaaccttgtcagattctgctgaggatgcaagtacggggaaggtggaattga<br>cccttgggtttggcagcagtaggtgtcagccatgagcctgacaatgtggtggagacaaaaaagccag<br>gtggggatgggctcaggggaggggagaggagcagtagaggtggcaagtgtggacggttcttctg<br>gagctttgctgttggagaggagggggtcattgttagcaacgataatagaataaagaggacttttatttt<br>attttattgttttgagacgaagtctcgctcttttcccataggctggagtgcaatggtgtgatctcagctcact<br>gcaacctctgcttcctgggtttaagtgattctcctgcctcagcctcccgagtagctgggattacaggtgac<br>tgccaccacgctcagctaagttttgtatttttagtagagacggggtttcaccatgttggccaggctggtct<br>cgaactcttgacctcaggtgatccgcccacctcggcctcccaaaatgctgggattacaggtgtaagcca<br>ccatgcccggcctaaagaggactttttaaggtagcaggtatgccagtaggctgcacagggatggaa<br>gtgatccattagaggagagatgatggagcagcaagaccatcaggagtgaagactccgtgacagagg<br>atgggacgcagtgcccaaggggaggttggccttgcagggaaagcacatgcctgtccctccctcataa<br>gcttcatttggacaaaacatgtaaaatccggtgtgttgtggaggccttttgattggggaactgtaacgct<br>gcctatcgagcaacagcactttaagcaggtggctttgttcaaattaaaggttcttctttttcttttcaggcat<br>catggaccgatctaaagaaaactgcatttcaggacctgttaaggtaaattgaataatctgtaatctcattc<br>acatttataaac**actagt**
pGL4.54[luc2/TK]	Promega, Madison, WI	E5061
pNL2.2[NlucP/Hygro]	Promega	N1071
AURKA-exon I (S)	Genewiz<br>**NheI** (**gctagc**)<br>and **HindIII (aagctt)**	**gctagc**accacttccgggttcttagggagcaagtgcgcctgcgcgcggtgtgcgcccttaaacgcg<br>actcaaggcgtcgggtttgttgtcaaccaatcacaaggcagcctcgctcgagcgcaggccaatcggc<br>tttctagctagagggtttaactcctatttaaaaagaagaacctttgaattctaacggctgagctcttgga<br>agacttgggtccttgggtcgcag**aagctt**
AURKA-exon I (S)+II	Genewiz	**gctagc**accacttccgggttcttagggagcaagtgcgcctgcgcgcggtgtgcgcccttaaacgcg<br>actcaaggcgtcgggtttgttgtcaaccaatcacaaggcagcctcgctcgagcgcaggccaatcggc<br>tttctagctagagggtttaactcctatttaaaaagaagaacctttgaattctaacggctgagctcttgga<br>agacttgggtccttgggtcgcaggctggagtgcaatggtgtgatctcagctcactgcaacctctgcttc<br>ctgggtttaagtgattctcctgcctcagcctcccgagtagctgggattacag**aagctt**

AURKA-exon I (S)+III	Genewiz	**gctagc**accacttccgggttcttagggagcaagtgcgcctgcgcgcggtgtgcgcccttaaacgcgactcaaggcgtcgggtttgttgtcaaccaatcacaaggcagcctcgctcgagcgcaggccaatcggctttctagctagagggtttaactcctatttaaaaagaagaacctttgaattctaacggctgagctcttggaagacttgggtccttgggtcgcaggctggagtgcaatggtgtgatctcagctcactgcaacctctgcttcctgggtttaagtgattctcctgcctcagcctcccgagtagctgggattacag**aagctt**
AURKA-exon I (S)+II+III	Genewiz	**gctagc**accacttccgggttcttagggagcaagtgcgcctgcgcgcggtgtgcgcccttaaacgcgactcaaggcgtcgggtttgttgtcaaccaatcacaaggcagcctcgctcgagcgcaggccaatcggctttctagctagagggtttaactcctatttaaaaagaagaacctttgaattctaacggctgagctcttggaagacttgggtccttgggtcgcagggtctcactccattgcccaggccagagtgcggggatatttgataagaaacttcagtgaaggccgggcgcggtggctcatgcccgtaatcccagcattttcggaggccgaggctggagtgcaatggtgtgatctcagctcactgcaacctctgcttcctgggtttaagtgattctcctgcctcagcctcccgagtagctgggattacag**aagctt**
AURKA-exon I (L)	Genewiz	**gctagc**accacttccgggttcttagggagcaagtgcgcctgcgcgcggtgtgcgcccttaaacgcgactcaaggcgtcgggtttgttgtcaaccaatcacaaggcagcctcgctcgagcgcaggccaatcggctttctagctagagggtttaactcctatttaaaaagaagaacctttgaattctaacggctgagctcttggaagacttgggtccttgggtcgcaggtgggagccgacgggtgggtagaccgtgggggatatctcagtggcggacgaggacggcggggacaaggggcggctggtcggagtggcggagcgtcaagtcccctgtcggttcctccgtccctgagtgtccttggcgctgccttgtgcccgcccagcgcctttgcatccgctcctgggcaccgaggcgccctgtaggatactgcttgttacttattacagctagag**aagctt**
AURKA-exon I (L)+II	Genewiz	**gctagc**accacttccgggttcttagggagcaagtgcgcctgcgcgcggtgtgcgcccttaaacgcgactcaaggcgtcgggtttgttgtcaaccaatcacaaggcagcctcgctcgagcgcaggccaatcggctttctagctagagggtttaactcctatttaaaaagaagaacctttgaattctaacggctgagctcttggaagacttgggtccttgggtcgcaggtgggagccgacgggtgggtagaccgtgggggatatctcagtggcggacgaggacggcggggacaaggggcggctggtcggagtggcggagcgtcaagtcccctgtcggttcctccgtccctgagtgtccttggcgctgccttgtgcccgcccagcgcctttgcatccgctcctgggcaccgaggcgccctgtaggatactgcttgttacttattacagctagagggtctcactccattgcccaggccagagtgcggggatatttgataagaaacttcagtgaaggccgggcgcggtggctcatgcccgtaatcccagcattttcggaggccgag**aagctt**
AURKA-exon I (L)+III	Genewiz	**gctagc**accacttccgggttcttagggagcaagtgcgcctgcgcgcggtgtgcgcccttaaacgcgactcaaggcgtcgggtttgttgtcaaccaatcacaaggcagcctcgctcgagcgcaggccaatcggctttctagctagagggtttaactcctatttaaaaagaagaacctttgaattctaacggctgagctcttggaagacttgggtccttgggtcgcaggtgggagccgacgggtgggtagaccgtgggggatatctcagtggcggacgaggacggcggggacaaggggcggctggtcggagtggcggagcgtcaagtcccctgtcggttcctccgtccctgagtgtccttggcgctgccttgtgcccgcccagcgcctttgcatccgctcctgggcaccgaggcgccctgtaggatactgcttgttacttattacagctagaggctggagtgcaatggtgtgatctcagctcactgcaacctctgcttcctgggtttaagtgattctcctgcctcagcctcccgagtagctgggattacag**aagctt**
AURKA-exon I (L)+II+III	Genewiz	**gctagc**accacttccgggttcttagggagcaagtgcgcctgcgcgcggtgtgcgcccttaaacgcgactcaaggcgtcgggtttgttgtcaaccaatcacaaggcagcctcgctcgagcgcaggccaatcggctttctagctagagggtttaactcctatttaaaaagaagaacctttgaattctaacggctgagctcttggaagacttgggtccttgggtcgcaggtgggagccgacgggtgggtagaccgtgggggatatctcagtggcggacgaggacggcggggacaaggggcggctggtcggagtggcggagcgtcaagtcccctgtcggttcctccgtccctgagtgtccttggcgctgccttgtgcccgcccagcgcctttgcatccgctcctgggcaccgaggcgccctgtaggatactgcttgttacttattacagctagagggtctcactccattgcccaggccagagtgcggggatatttgataagaaacttcagtgaaggccgggcgcggtggctcatgcccgtaatcccagcattttcggaggccgaggctggagtgcaatggtgtgatctcagctcactgcaacctctgcttcctgggtttaagtgattctcctgcctcagcctcccgagtagctgggattacag**aagctt**

Restriction Enzymes & Cloning	Company	Catalog #
PstI	New England BioLabs	R3140S
SalI	New England BioLabs	R3138S
SpeI	New England BioLabs	R3133S
FastDigest NheI	Thermo Fisher Scientific	FD0974
FastDigest HindIII	Thermo Fisher Scientific	FD0505
T4 DNA Ligase	New England BioLabs	M0202S
T4 Polynucleotide Kinase	New England BioLabs	M0201S
Quick CIP	New England BioLabs	M0525S
One Shot MAX Efficiency DH5α-T1R Competent Cells	ThermoFisher Scientific	12297016

Antibodies/Stain	Source	Catalog #	Assay Concentration
Rabbit anti-AURKA	Cell Signaling Technologies, Danvers, MA	14475	Immunoblot 1:500
Rabbit anti-HNRNPH	Bethyl Laboratories, Montgomery, TX	A300-511A	Immunoblot 1:1500
Mouse anti-VINCULIN	Santa Cruz Biotechnology, Dallas, TX	sc-25336	Immunoblot 1:1500
Rabbit anti-ACTIN	Cell Signaling Technologies,	4967	Immunoblot 1:1500
Rabbit anti-GAPDH	Abcam	ab8245	Immunoblot 1:1500
Rabbit anti-N-MYC	Cell Signaling Technologies	9405S	Immunoblot 1:1000
Goat Anti Rabbit IgG-HRP	Cell Signaling Technologies	7074S	Immunoblot 1:3000
Goat Anti Mouse IgG-HRP	Cell Signaling Technologies	7076S	Immunoblot 1:3000

siRNAs	Company	Catalog #	Sequence
siNeg	Qiagen, Germantown, MD	SI03650318	-
siHNRNPH1 (s6730)	Ambion (Thermo Fisher Scientific)	s6730	5'-GGAUUUGGGUCAGAUAGAUTT-3'
siHNRNPH1 (s6728)	Ambion (Thermo Fisher Scientific)	s6728	5'-GGAAGCAUACUGGUCCAAAUTT-3'

Chemicals and other resources	Company	Catalog #
Fetal Bovine Serum	Invitrogen (Thermo Fisher Scientific)	A56708-01
DMEM	Invitrogen (Thermo Fisher Scientific)	11995-065
EMEM	ATCC	30-2003
RPMI-1640	Invitrogen (Thermo Fisher Scientific)	11875-093
DPBS	Invitrogen (Thermo Fisher Scientific)	10010-023
0.25% Trypsin-EDTA	Invitrogen (Thermo Fisher Scientific)	25200-056
MycoAlert Mycoplasma Detection Kit	Lonza, Walkersville, MD	LT07-218
Plasmocin	InvivoGen USA, San Diego, CA	ant-mpp
Lipofectamine RNAiMAX	Invitrogen (Thermo Fisher Scientific)	13778100
Lipofectamine 2000	Invitrogen (Thermo Fisher Scientific)	11668500
OptiMEM	Invitrogen (Thermo Fisher Scientific)	31985-070
Maxwell 16 LEV simplyRNA purification kit	Promega, Madison, WI	AS1390
Nano-Glo Dual-Luciferase Reporter Assay System	Promega	N1610
Actinomycin D	Sigma, St. Louis, MO	A1410
DMSO	Invitrogen (Thermo Fisher Scientific)	D12345
QIAquick Gel Extraction Kit	Qiagen	28704
QIAquick PCR Purification Kit	Qiagen	28104
PureLink HiPure Plasmid Midiprep Kit	Invitrogen (Thermo Fisher Scientific)	K210005
PureLink HQ Mini Plasmid Purfication Kit	Invitrogen (Thermo Fisher Scientific)	2385512
Cell Extraction Buffer	Invitrogen (Thermo Fisher Scientific)	FNN0011
Pierce BCA Protein Assay Kit	Thermo Fisher Scientific	A55864
Poly-L-Lysine	Millipore Sigma, Burlington, MA	P8920
PureCol	Advanced Biomatrix, Carlsbad, CA	5005

Triton-X 100	Millipore Sigma	X100
NuPAGE™ Bis-Tris Mini Protein Gels, 4–12%	Thermo Fisher Scientific	NP0322BOX
NuPAGE™ MOPS SDS Running Buffer (20X)	Thermo Fisher Scientific	NP0001
PageRuler™ Prestained Protein Ladder, 10 to 180 kDa	Thermo Fisher Scientific	26616
Cell Line Nucleofector® Kit V	Lonza	VCA-1003
Cell Line Nucleofector® Kit L	Lonza	VCA-1005
Cell Line Nucleofector® Kit R	Lonza	VCA-1001

**Software, R packages, and other tools**	
GraphPad Prism version 10.2.3	https://www.graphpad.com/
Microsoft Excel (Mac Version 16.95.4)	https://www.microsoft.com/en-us/microsoft-365/excel
CCBR pipeliner Renee	https://ccbr.github.io/RENEE/latest/#renee---rna-sequencing-analysis-pipeline
R/R studio	https://www.r-project.org/ https://posit.co/download/rstudio-desktop/
ImageJ	https://imagej.net/software/fiji/
rMATS	https://github.com/Xinglab/rmats-turbo
Maser	https://bioconductor.org/packages/release/bioc/html/maser.html
Metascape	https://metascape.org/gp/index.html#/main/step1
MAJIQ and MAJIQ-L	https://majiq.biociphers.org/
MAJIQlopedia	https://majiq.biociphers.org/majiqlopedia/
EBSeq	https://bioconductor.org/packages/release/bioc/html/EBSeq.html
IsoSeq4	https://isoseq.how/
Target/TCGA data: UCSC Xena	https://xenabrowser.net/

**PCR Conditions**			
***TCF3*-exon 18 Splice Assay PCR (2 µL cDNA; Phusion GC)**			
98°C for 30 sec			
98°C for 10 sec	35 cycles		
64°C for 10 sec			
72°C for 30 sec			
72°C for 5 min			
***EIF4G1* (exon 1 to exon5 PCR; 2 µL cDNA; Phusion GC)**			
98°C for 30 sec			
98°C for 8 sec	35 cycles		
66°C for 12 sec			
72°C for 15 sec			
72°C for 5 min			
***EIF4G1* (exon 4/µexon PCR; 2 µL cDNA: Phusion GC)**			
98°C for 30 sec			
98°C for 8 sec	35 cycles		
65°C for 12 sec			
72°C for 15 sec			
72°C for 5 min			
***AURKA* and 18s (endogenous transcript variants; 1 µL cDNA; Q5 Hot Start)**			
98°C for 30 sec			
98°C for 8 sec	30 cycles		
65°C for 20 sec			
72°C for 8 sec			
72°C for 2 min			
**AURKA Minigene**			
Junction specific forward primer	cDNA (µL)	Annealing temperature (°C)	# of Cycles
Ia+II	8	65	35
Ia+III	8	66	35

Ia+CDS1	8	66	35
Ib+II	4	64	32
Ib+III	4	65	32
Ib+CDS1	4	66	32
Ic+II	4	64	32
Ic+III	4	65	32
Ic+CDS1	4	65	32

Tumor data extracted from MAJIQlopedia (5)		
Abbreviation	Tumor type	Sample number and original data source
ACC	Adrenocortical carcinoma	n=153, TCGA
BLCA	Bladder urothelial carcinoma	n=432, TCGA
BRCA	Breast invasive carcinoma	n=498, TCGA
CCSK	Clear Cell Sarcoma of the Kidney	n=13, TARGET
CESC	Cervical squamous cell carcinoma and endocervical adenocarcinoma	n=309, TCGA
CHOL	Cholangiocarcinoma	n=35, TCGA
COAD	Colon adenocarcinoma	n=352, TCGA
ESCA	Esophageal carcinoma	n=181, TCGA
GBM	Glioblastoma multiforme	n=158, TCGA
HNSC	Head and neck squamous cell carcinoma	n=565, TCGA
KICH	Kidney chromophobe	n=155, TCGA
KIRC	Kidney renal clear cell carcinoma	n=298, TCGA
KIRP	Kidney renal papillary cell carcinoma	n=323, TCGA
LGG	Brain lower grade glioma	n=499, TCGA
LIHC	Liver hepatocellular carcinoma	n=423, TCGA
LUAD	Lung adenocarcinoma	n=500, TCGA
LUSC	Lung squamous cell carcinoma	n=496, TCGA
MESO	Mesothelioma	n=166, TCGA
NB	Neuroblastoma	n=168, TARGET
OV	Ovarian Serous cystadenocarcinoma	n=401, TCGA
PCPG	Pheochromocytoma and Paraganglioma	n=187, TCGA
PAAD	Pancreatic adenocarcinoma	n=183, TCGA
PRAD	Prostate adenocarcinoma	n=500, TCGA
READ	Rectum adenocarcinoma	n=104, TCGA
SARC	Sarcoma	n=263, TCGA
SKCM	Skin cutaneous melanoma	n=473, TCGA
STAD	Stomach adenocarcinoma	n=374, TCGA
TGCT	Testicular germ cell tumors	n=139, TCGA
THCA	Thyroid carcinoma	n=500, TCGA
UCEC	Uterine corpus endometrial carcinoma	n=167, TCGA
UCS	Uterine carcinosarcoma	n=111, TCGA
UVM	Uveal melanoma	n=79, TCGA
WT	Wilms tumors	n=130, TARGET

References

1. Neckles, C., Boer, R.E., Aboreden, N., Cross, A.M., Walker, R.L., Kim, B.H., Kim, S., Schneekloth, J.S., Jr. and Caplen, N.J. (2019) HNRNPH1-dependent splicing of a fusion oncogene reveals a targetable RNA G-quadruplex interaction. *RNA*, **25**, 1731-1750.
2. Yamazaki, T., Liu, L., Lazarev, D., Al-Zain, A., Fomin, V., Yeung, P.L., Chambers, S.M., Lu, C.W., Studer, L. and Manley, J.L. (2018) TCF3 alternative splicing controlled by hnRNP H/F regulates E-cadherin expression and hESC pluripotency. *Genes Dev*, **32**, 1161-1174.
3. Gonatopoulos-Pournatzis, T., Niibori, R., Salter, E.W., Weatheritt, R.J., Tsang, B., Farhangmehr, S., Liang, X., Braunschweig, U., Roth, J., Zhang, S. *et al.* (2020) Autism-Misregulated eIF4G Microexons Control Synaptic Translation and Higher Order Cognitive Functions. *Mol Cell*, **77**, 1176-1192 e1116.
4. Singh, G. and Cooper, T.A. (2006) Minigene reporter for identification and analysis of cis elements and trans factors affecting pre-mRNA splicing. *Biotechniques*, **41**, 177-181.
5. Quesnel-Vallieres, M., Jewell, S., Lynch, K.W., Thomas-Tikhonenko, A. and Barash, Y. (2024) MAJIQlopedia: an encyclopedia of RNA splicing variations in human tissues and cancer. *Nucleic Acids Res*, **52**, D213-D221.
6. Vaquero-Garcia, J., Aicher, J.K., Jewell, S., Gazzara, M.R., Radens, C.M., Jha, A., Norton, S.S., Lahens, N.F., Grant, G.R. and Barash, Y. (2023) RNA splicing analysis using heterogeneous and large RNA-seq datasets. *Nat Commun*, **14**, 1230.

Figure S1

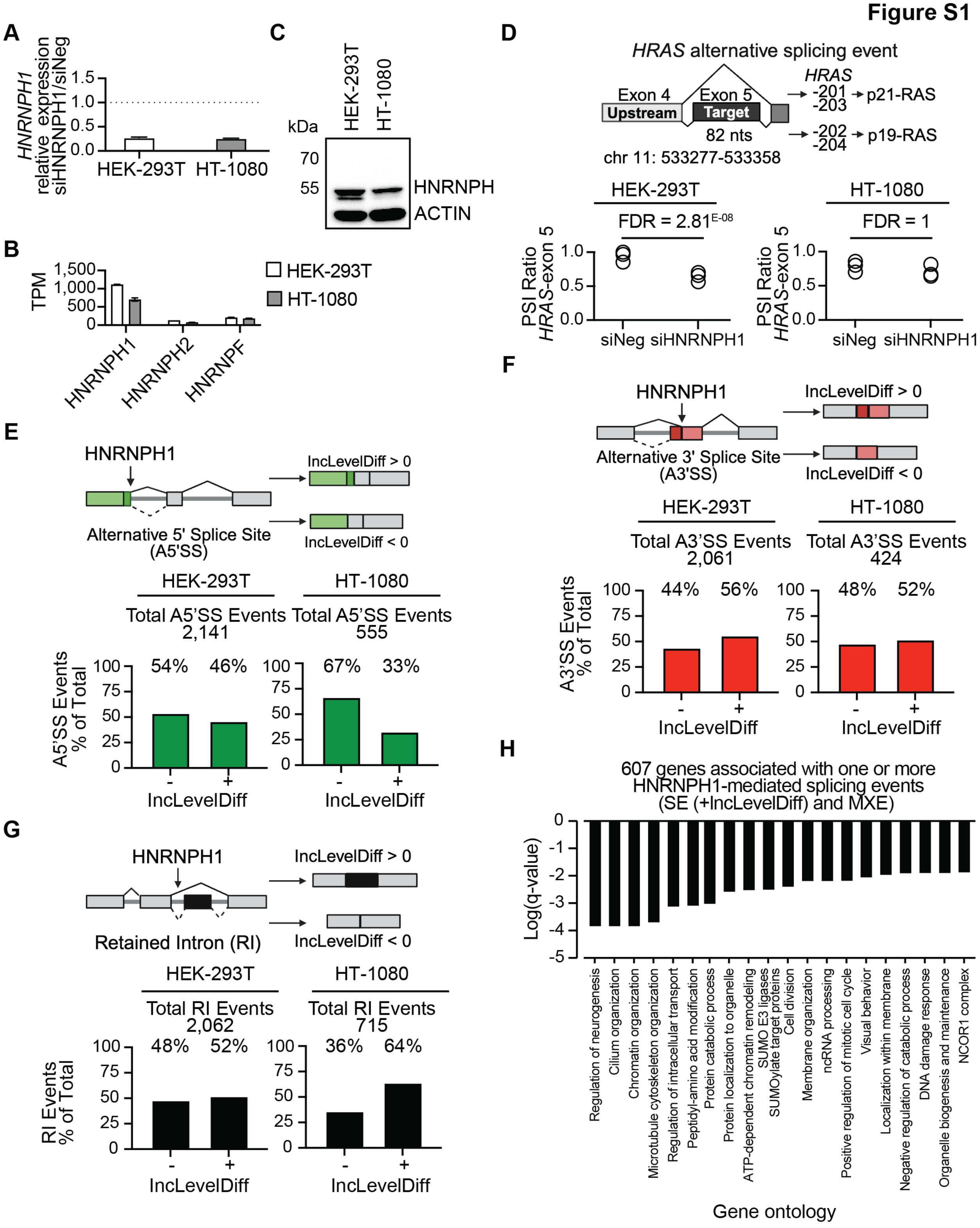

Supplementary Figure S1. HNRNPH1's transcriptome-wide regulation of alternative splicing (**A**) PCR-based quantification of *HNRNPH1* expression in siHNRNPH1-transfected HEK-

293T or HT-1080 cells normalized to siNeg-transfected cells. *HNRNPH1* expression normalized to *NACA*. (**B**) RNA-seq read counts for *HNRNPH1* and *HNRNPH2* in HEK-293T and HT-1080 cells. (**C**) Immunoblot analysis of whole cell lysates prepared from the indicated cell lines and probed using an antibody that due to the homology of HNRNPH1 and HNRNPH2 cannot distinguish between these proteins and an antibody against ACTIN as a loading control. Data shown is representative of three replicates. (**D**) A schematic of the *HRAS*-exon 5 splicing event that defines expression of transcript variants encoding the p21 and p19-HRAS isoforms and the rMATS analysis of the inclusion of the *HRAS*-exon 5 in HEK-293T and HT-1080 cells treated as indicated. (**E**) A schematic summarizing IncLevelDiff used to define HNRNPH1-dependent splicing events that make use of alternative 5′ splice sites and the percent distribution of IncLevelDiff values per cell line. (**F**) A schematic summarizing IncLevelDiff used to define HNRNPH1-dependent splicing events that make use of alternative 3′ splice sites and the percent distribution of IncLevelDiff values per cell line. (**G**) A schematic summarizing IncLevelDiff used to define HNRNPH1-dependent splicing events involving retained intron (RI) events and the percent distribution of IncLevelDiff values per cell line. (**H**) Gene ontology (GO) analysis of the 607 genes associated with one or more HNRNPH1-mediated splicing events (SE event, positive IncLevelDiff) and MXE events. Enrichment analysis performed using Metascape.

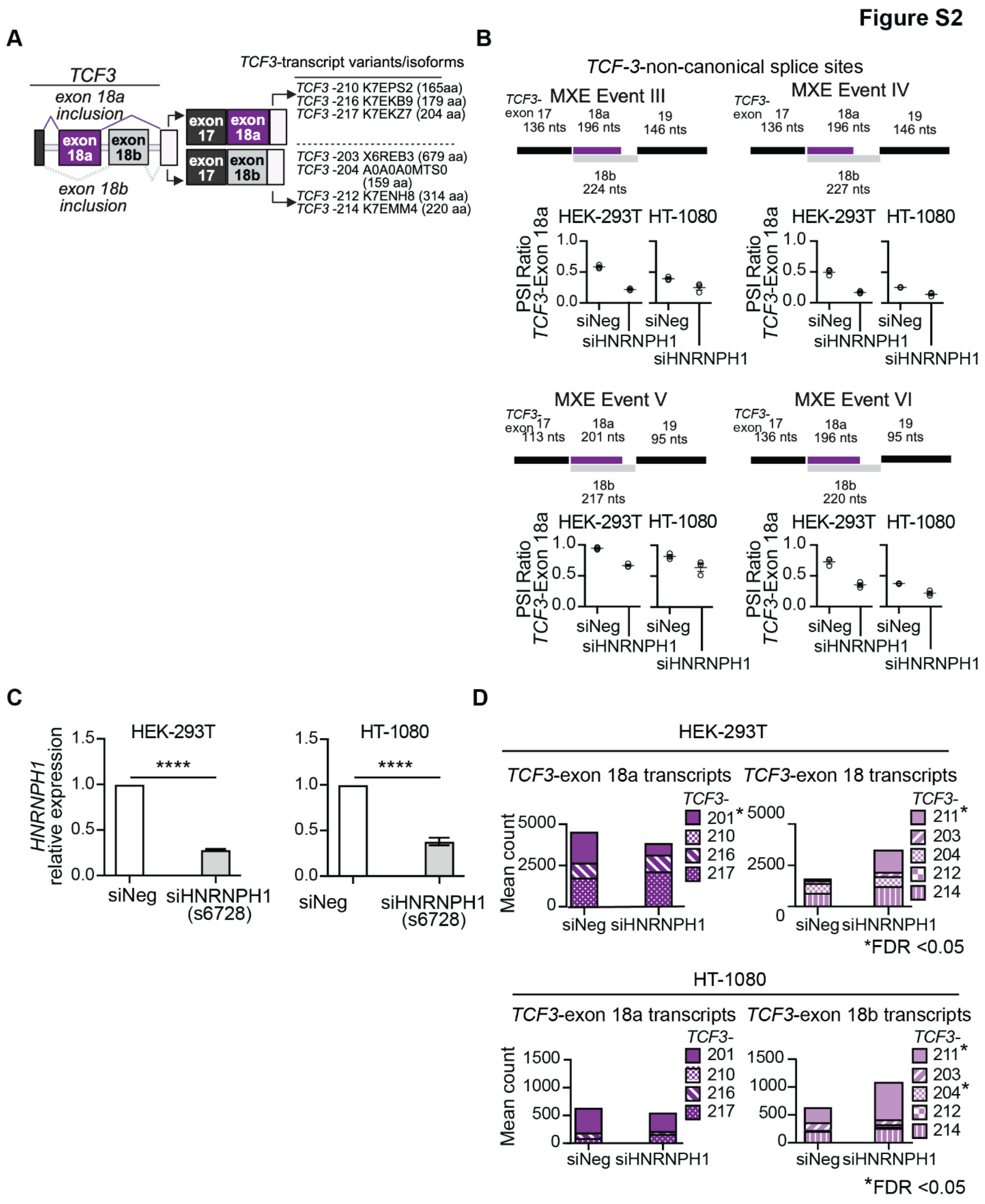

Supplementary Figure S2: HNRNPH1's regulation of a *TCF3* mutually exclusive splicing event (**A**) Schematic of *TCF3*-exon 18a and *TCF3*-exon 18b containing less abundant transcript

variants and their encoded protein isoforms. (**B**) rMATS outputs for the *TCF3*-exon 18a and -exon 18b MXE splicing events that use non-canonical splice sites in the indicated cell lines transfected with the indicated siRNAs (three experimental replicas (open circles) and mean and SEM (lines). (**C**) qRT-PCR analysis of *HNRNPH1* expression in control (siNeg) and siHNRNPH1(s6728)-transfected HEK-293T and HT-1080 cells (unpaired t test with Welch's correction, P value **** <0.0001). (**D**) Quantification of *TCF3*-exon 18a or -exon 18b containing transcript variants expressed in control (siNeg) or HNRNPH1-depleted (siHNRNPH1) HEK-293T or HT-1080 cells (EBSeq). * Indicates FDR <0.05.

Figure S3

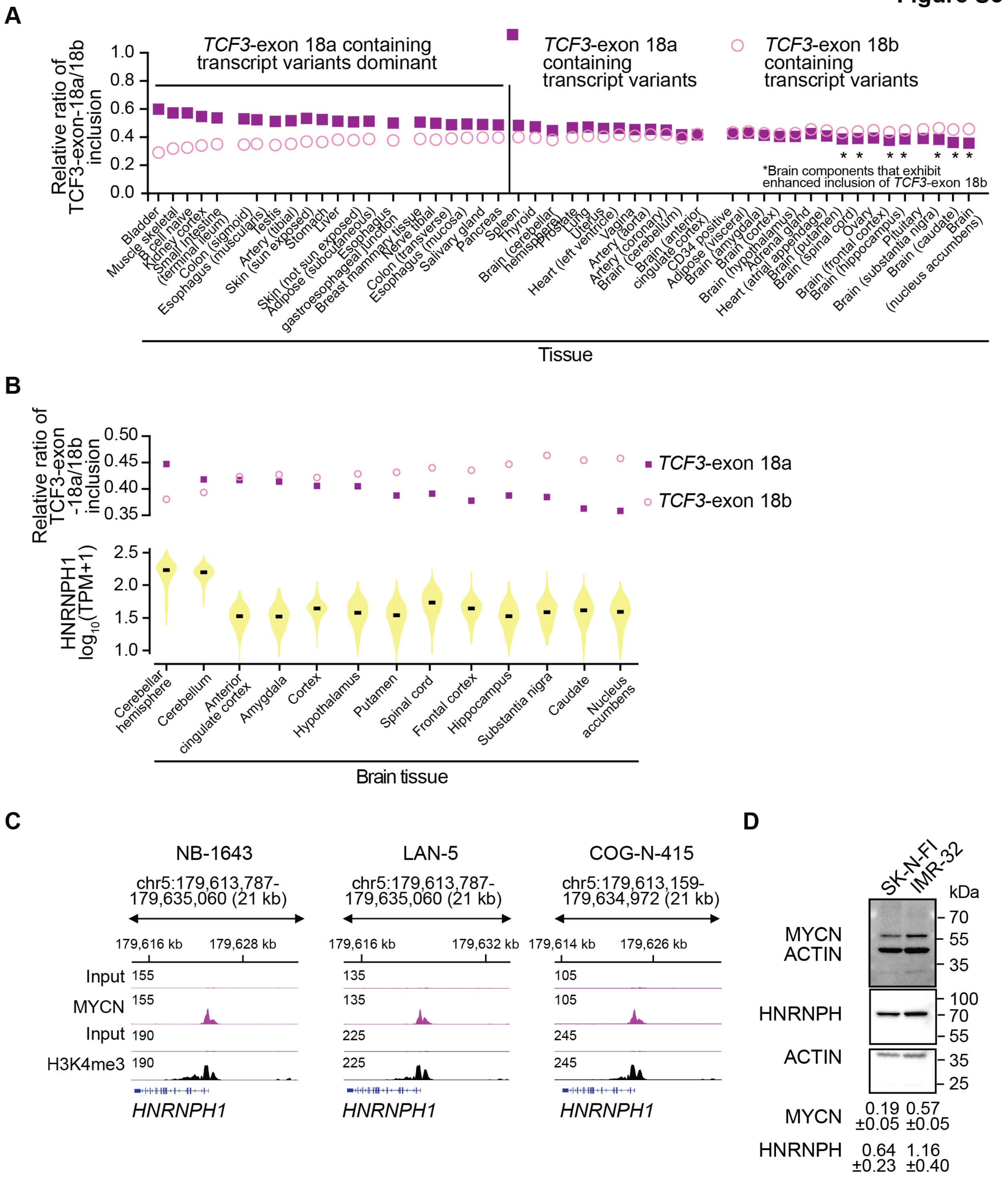

Supplementary Figure S3: The differential expression of *TCF3* transcript variants in neuroblastoma (**A**) Ranked relative ratios of *TCF3*-exon 18a and *TCF3*-exon 18b usage in the indicated tissues. Data extracted from MAJIQlopedia. (**B**) Ranked relative ratios of *TCF3*-exon 18a

and *TCF3*-exon 18b usage and *HNRNPH1* expression in the indicated brain tissues (black bars indicate the median). Data extracted from MAJIQlopedia and GTEx portal, respectively (**C**) The *HNRNPH1* locus of NB-143, LAN-5 and COG-N-145 NB cells showing MYCN binding and H3K4me3 deposition (**D**) Immunoblot analysis of whole cell lysates extracted from the indicated NB cell lines probed using antibodies against MYCN and HNRNPH. Relative intensities (normalized to ACTIN) are shown on the bottom of each lane).

Figure S4

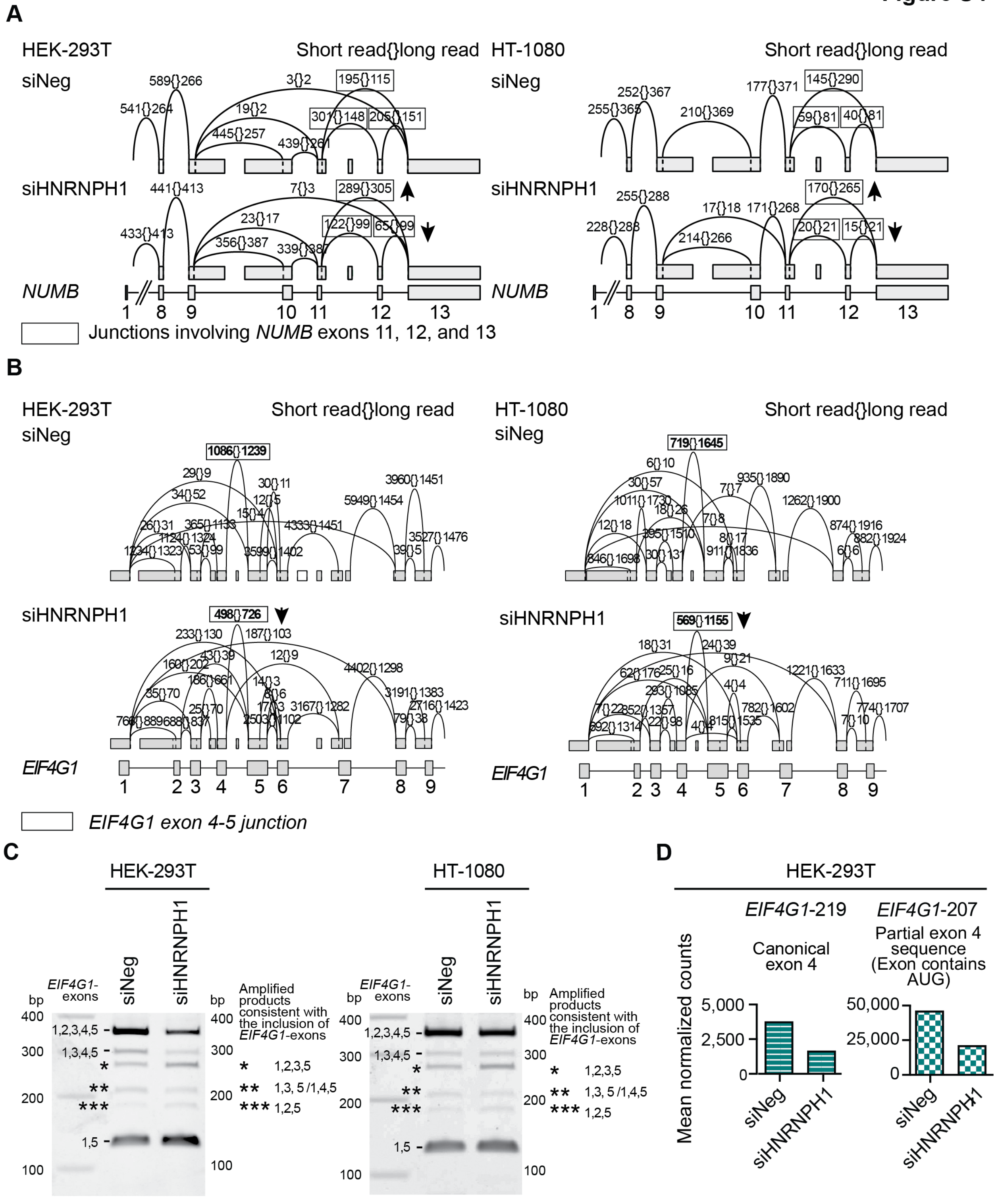

Supplementary Figure S4: HNRNPH1 regulates the inclusion of *NUMB* exon 12 and usage of the *EIF4G1* exon 4-5 junction (**A**) Visualization and quantification of splicing events focused

on the 3′ *NUMB* exons (exons 9-13) expressed in HEK-293T and HT-1080 cells. The number on the left of each {} shows the median read count from the short-read RNA-seq data and the number on the right, the sum of full-length counts from the long-read data. The black rectangles indicate junctions involving NUMB exons 11, 12, and 13, and the arrows indicate the direction of change following the silencing of *HNRNPH1*. Data assessed using the VOILA visualization tool and quantified using the HET (heterogen) quantifier within MAJIQ (6). (**B)** Visualization and quantification of splicing events involving the first 9 exons of *EIF4G1* expressed in HEK-293T cells. The number on the left of each {} shows the median read count from the short-read RNA-seq data and the number on the right the sum of full-length counts from the long-read data. The black rectangles indicate the *EIF4G1* exon 4-5 junctions and arrows the direction of change following the silencing of *HNRNPH1*. Data assessed using the VOILA visualization tool and quantified using the HET quantifier within MAJIQ. (**C**) Representative longer exposures of the PCR-based splice assay designed to detect the usage of *EIF4G1* exons 1 – 5 in HEK-293T or HT-1080 cell lines transfected as indicated. The *, **, and *** specify amplified products predicted to consist of the indicated *EIF4G1* exons. (**D**) The mean normalized counts of the indicated *EIF4G1* transcript variants in control (siNeg) and HNRNPH1-depleted (siHNRNPH1) HEK-293T (EBSeq).

Figure S5

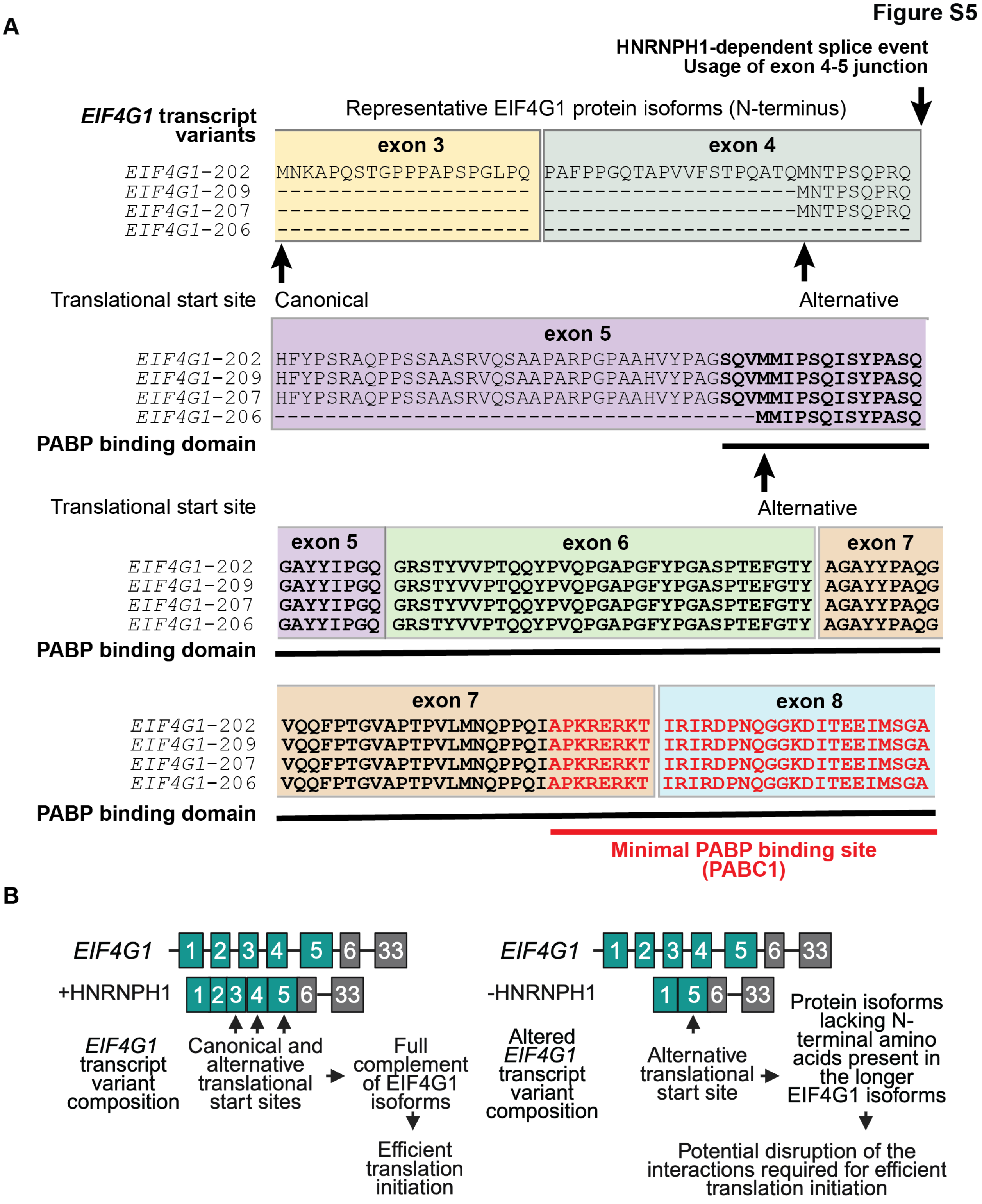

Supplementary Figure S5: The depletion of HNRNPH1 reduces *EIF4G1* transcript variant composition (**A**) The amino acid sequences of EIF4G1 isoform N-terminus regions encoded by

the indicated transcript variants organized relative to the full-length EIF4G1 protein. The indicated exon annotations refer to the full-length *EIF4G1*-202 transcript variant. Information shown gathered from Ensembl gene ID ENSG00000114867 and UniProt entry Q04637. (**B**) A model illustrating HNRNPH1's contribution to the maintenance of EIF4G1 isoform composition through its regulation of the exon4/exon 5 splice event and the concept that HNRNPH1-depletion will alter EIF4G1 isoform composition by promoting the expression of isoforms lacking the amino acids which form the N-terminus of longer EIF4G1 isoforms. We speculate that this shift in isoform composition could disrupt the interactions required for efficient translation initiation.

Figure S6

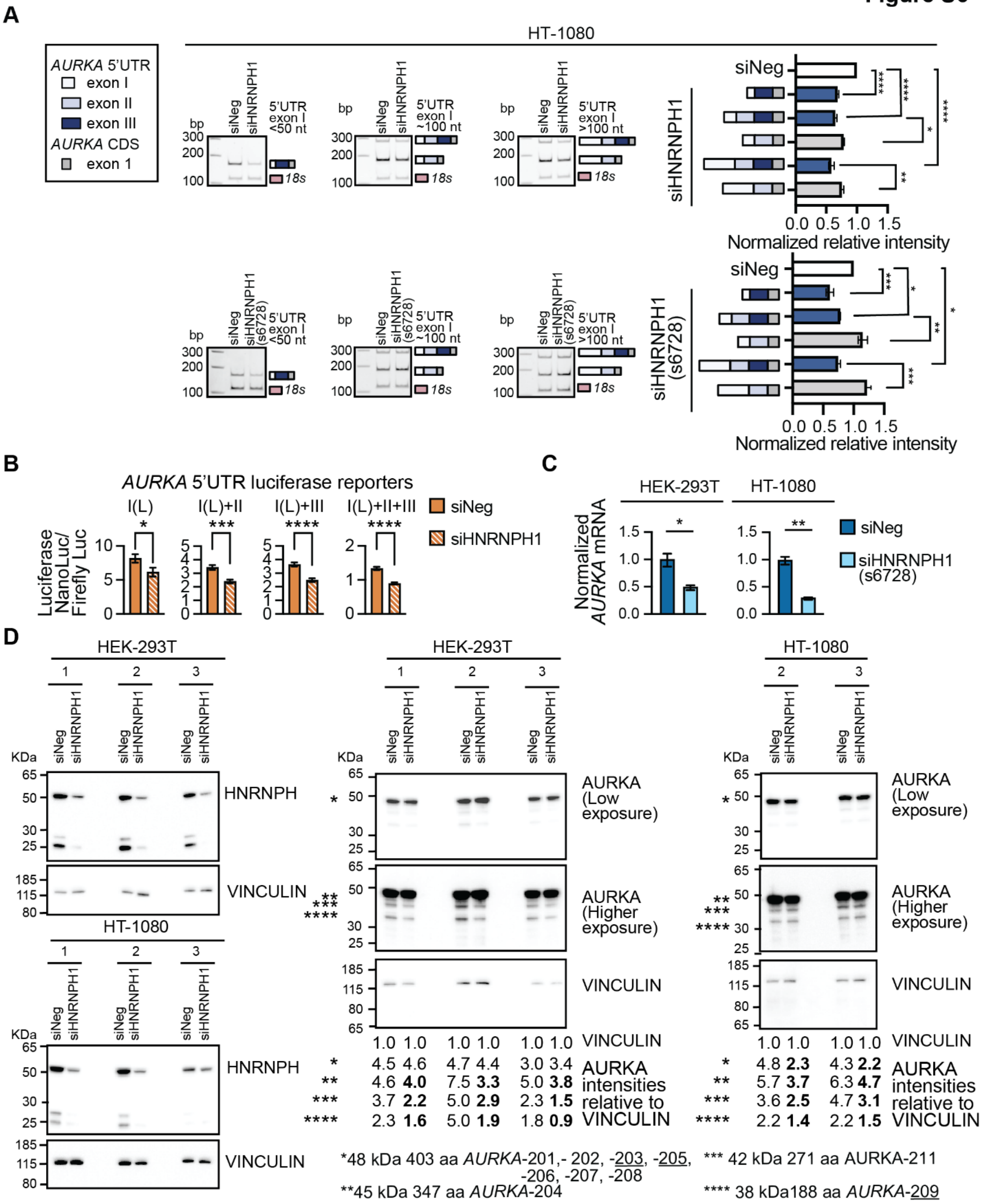

Supplementary Figure S6: HNRNPH1's regulation of an *AURKA* 5'UTR skipped exon (SE) event and *AURKA* RNA stability. (**A**) qPCR-based analysis of *AURKA* transcript variants that

include *AURKA*-5'UTR-exon III in transfected HT-1080 cells (siNeg and siHNRNPH1: upper panels; siNeg and siHNRNPH1/s6278: lower panels), and quantification of the amplified products normalized to the intensity of the 18S amplified product (mean±SEM of three biological replicates). Statistical analysis: ordinary one-way ANOVA, P values *** <0.001, **** <0.0001. (**B**) Relative luciferase expression (NanoLuc/Firefly) 48 hours post-transfection of HEK-293T cells with the indicated reporter construct and the indicated siRNA (mean ± SEM n=6 per condition). Statistical analysis: unpaired t test with Welch's correction, P values * <0.05, *** <0.001, **** <0.0001. (**C**) qRT-PCR analysis of *AURKA* expression (*RPL27* normalized) following the silencing of *HNRNPH1*; unpaired t test with Welch's correction, P * <0.05, P ** <0.01. (**D**) The immunoblot analysis of whole cell lysates prepared from siNeg or siHNRNPH1(s6728)-transfected HEK-293T or HT-1080 cells probed using antibodies against the indicated protein. The images show the results of the three independent transfections per siRNA performed using HEK-293T cells and the two independent transfections per siRNA performed in HT-1080 cells. **Figure 7H** shows the results of the third transfection conducting using HT-1080 cells. The asterisks indicate the detected AURKA protein isoform and the values indicate the VINCULIN normalized quantification of each isoform.
